# Intra-Articular Ozone Injections for Temporomandibular Joint Disorders: A Systematic Review

**DOI:** 10.3390/jcm15082955

**Published:** 2026-04-13

**Authors:** Wojciech Macek, Maciej Chęciński, Amelia Hoppe, Kamila Chęcińska, Klaudia Kwiatkowska, Paweł Sikora, Karolina Lubecka, Filip Bliźniak, Kacper Galant, Kalina Romańczyk, Maciej Sikora

**Affiliations:** 1 Department of Oral Surgery, Preventive Medicine Center, Komorowskiego 12, 30-106 Cracow, Poland; ld.wojciech.macek@wp.pl (W.M.); amelia.a.hoppe@gmail.com (A.H.); klaudia011998@wp.pl (K.K.); lubeckarolina@gmail.com (K.L.); kalina.romanczyk@wp.pl (K.R.); 2 National Medical Institute of the Ministry of the Interior and Administration, Wołoska 137 Str., 02-507 Warsaw, Poland; sikora-maciej@wp.pl; 3 Department of Maxillofacial Surgery, Hospital of the Ministry of Interior, Wojska Polskiego 51, 25-375 Kielce, Poland; 4 Faculty of Medicine, Medical University of Lublin, Al. Racławickie 1, 20-059 Lublin, Poland; pawelsikora2205@gmail.com; 5 Department of Maxillofacial Surgery, Rydygier Hospital, Osiedle Złotej Jesieni 1, 31-826 Kraków, Poland; fblizniak@gmail.com; 6 Faculty of Medicine, Medical University of Lodz, Al. Kościuszki 4, 90-419 Lodz, Poland; kacpergalant.ld@gmail.com; 7 Department of Biochemistry and Medical Chemistry, Pomeranian Medical University, 72 Powstańców Wielkopolskich Street, 70-111 Szczecin, Poland

**Keywords:** ozone therapy, pain management, randomized controlled trials as topic, temporomandibular joint, temporomandibular joint disorders

## Abstract

**Objectives:** Temporomandibular joint (TMJ) disorders are an increasingly common problem affecting patients. This systematic review aimed to analyze the available literature regarding the effectiveness of intra-articular ozone gas injections and their effects on pain reduction and jaw mobility. **Methods:** The review was conducted in accordance with PRISMA 2020 guidelines. Bielefeld Academic Search Engine, Cochrane Library, Google Scholar, PubMed, and references were searched (October 2025). Randomized controlled trials evaluating intra-TMJ gas injection compared to other intra-articular administration were included in the review. Analyses were performed both within and between groups. Risk of bias was assessed using the Cochrane RoB-2 tool, and the certainty of evidence was assessed using the GRADE approach. **Results:** Five studies out of 180, including a total of 230 patients, were included in the review. The studies reported using ozone at concentrations of 10–30 μg/mL. All included studies reported reductions in pain and improvements in mandibular mobility after treatment. In some comparisons, ozone-based interventions showed more favorable outcomes than control interventions; however, the findings were not consistent across outcomes, and the certainty of evidence was limited. **Conclusions:** Current evidence is insufficient to determine the effectiveness of intra-articular ozone injections for TMJ disorders. Some studies suggest possible short-term benefits in pain reduction and jaw mobility; however, the evidence is limited by high risk of bias, heterogeneity in treatment protocols, and very low certainty, particularly for longer-term outcomes. More rigorous and standardized randomized trials are required.

## 1. Introduction

Temporomandibular disorders (TMDs) comprise a heterogeneous group of structural and functional abnormalities involving the temporomandibular joints (TMJs), masticatory muscles, and associated components of the stomatognathic system. They represent one of the most common causes of orofacial pain not directly related to dental pathology and are recognized as a significant global health problem, affecting up to one-third of the adult population [1]. The proper function of the temporomandibular joint depends on the integrity of both anatomical and neuromuscular components; disruption of either may adversely affect speech, mastication, and overall quality of life.

The etiology of TMD is multifactorial and involves the complex interaction of biomechanical, psychosocial, and physiological factors. Biomechanical contributors include parafunctional activities such as bruxism and clenching, abnormal joint loading, and occlusal interferences leading to repetitive microtrauma and intra-articular changes. Psychosocial factors, including stress, anxiety, and altered pain coping mechanisms, further contribute to symptom development and persistence. Maxillofacial trauma is also a significant etiological factor in temporomandibular joint disorders. Maxillofacial fractures (MFFs) can lead to post-traumatic changes in the biomechanics of the jaw and stress on the temporomandibular joint, resulting in chronic pain, functional limitations, and reduced quality of life, particularly in the early stages of recovery [2]. In addition, increasing attention has been directed toward altered pain processing and modulation pathways, which may explain symptom chronicity in a subset of patients. This multifactorial background complicates both diagnosis and treatment, often necessitating individualized and multimodal therapeutic strategies [1,3,4].

Clinically, patients with TMD may present with a wide spectrum of symptoms. The most frequently reported manifestations include pain localized to the TMJ region, the preauricular area, or the masticatory muscles, as well as joint sounds such as clicking or crepitus during mandibular movements. As the condition progresses, patients may develop functional limitations, including reduced mandibular mobility, difficulties with chewing and speaking, and pain radiating to the head and neck. Symptom exacerbation is commonly associated with periods of increased stress or excessive joint loading [4].

Given the complexity of TMD pathophysiology, management typically follows a multidisciplinary approach. Conservative treatment options include physiotherapy aimed at improving muscle function and joint mobility, occlusal therapy with stabilizing splints, behavioral interventions, and pharmacological management using analgesics, muscle relaxants, and nonsteroidal anti-inflammatory drugs (NSAIDs) [5,6]. Although these approaches are effective in many cases, conservative therapy may be insufficient in patients with chronic, recurrent, or treatment-resistant symptoms.

In recent years, minimally invasive interventions, particularly intra-articular injection therapies, have gained increasing popularity as alternative treatments for TMD. The effects of intra-articular therapies are often assessed in terms of pain reduction, jaw mobility, and quality of life. Recent papers indicate that arthrocentesis followed by intra-articular administration of medications such as platelet-rich fibrin, platelet-rich plasma, hyaluronic acid, nonsteroidal anti-inflammatory drugs, or hypertonic dextrose may provide clinical benefits, although differences between therapeutic methods remain limited and context-dependent [7,8]. Gases such as oxygen–ozone mixtures (O_2_–O_3_) are also used in intra-articular therapies. Among these, ozone therapy has emerged as a promising approach due to its dose-dependent biological effects and increasing use in chronic inflammatory conditions. Ozone has been shown to reduce inflammation, accelerate tissue regeneration, improve microcirculation and oxygenation, and modulate oxidative stress in treated tissues [9,10].

Upon contact with biological fluids, ozone generates reactive oxygen and nitrogen species (RONS) and lipid oxidation products (LOPs). At low, therapeutic concentrations, ozone induces a controlled oxidative stimulus that activates endogenous antioxidant and cytoprotective mechanisms [11,12]. A key pathway involved in this process is the Nrf2–Keap1 signaling axis. Mild oxidative stress promotes dissociation of Nrf2 from Keap1, allowing its translocation into the nucleus, where it activates the antioxidant response element (ARE) and upregulates the expression of protective enzymes such as heme oxygenase-1 (HO-1) and superoxide dismutase (SOD) [12,13]. Concurrently, activation of Nrf2 and related pathways leads to inhibition of NF-κB signaling, resulting in decreased expression of proinflammatory cytokines, including IL-1β, TNF-α, and IL-6, and reduced expression of inflammatory mediators and chemokines [13,14].

Beyond its anti-inflammatory effects, ozone has been shown to influence cartilage metabolism and immune responses. Experimental studies indicate that ozone may inhibit cytokine-induced matrix metalloproteinases (MMPs), particularly MMP-3 and MMP-13, thereby slowing the degradation of type II collagen and proteoglycans in articular cartilage. This chondroprotective mechanism has been demonstrated in osteoarthritis models and may also be relevant for TMJ-related degenerative lesions [15,16]. In addition, ozone has been reported to enhance chondrocyte survival and reduce apoptosis through modulation of autophagy-related pathways, including PPARγ and mTOR signaling. Ozone may also promote a phenotypic shift in macrophages from a pro-inflammatory to a regenerative profile, further supporting tissue repair processes [13].

Based on these biological properties, intra-articular ozone injections have been proposed as a potential therapeutic option for internal TMJ disorders, particularly those associated with disc displacement. Clinical studies suggest that O_2_–O_3_ injections may reduce pain and improve mandibular mobility and may serve as an alternative to nonsteroidal anti-inflammatory drugs and muscle relaxants [17]. Some reports indicate improved maximal interincisal opening (MIO) and pain reduction compared with control interventions [18]. Systematic reviews and meta-analyses have further suggested beneficial effects of ozone therapy on pain and jaw function in TMD patients; however, the low level of evidence and considerable risk of bias highlight the need for cautious interpretation of these findings [19].

To date, several systematic reviews have addressed the role of ozone therapy in TMD management [19,20,21]. However, recently published randomized controlled trials (RCTs) provide new data that allow for a renewed and more precise assessment of the effectiveness of intra-articular ozone therapy in this patient population.

The present systematic review aims to comprehensively assess and critically synthesize the available literature on intra-articular gas injections for the treatment of TMDs, with particular emphasis on clinical outcomes related to pain, mandibular mobility, and quality of life. The null hypothesis tested in this review is that intra-articular gas injections do not result in significant pain reduction or functional improvement compared with control interventions.

## 2. Material and Methods

### 2.1. Protocol and Registration

This systematic review was conducted according to the PRISMA 2020 guidelines [22]. The review protocol was prospectively registered in the PROSPERO database (registration number: CRD420251168037) [23]. The deviations from the protocol were:

- The omission of Scopus searches due to the lack of publicly available free access (reproducibility limitation).
- The addition of Google Scholar searches, which was not originally planned.
- An initially unplanned snowball reference search.

### 2.2. Eligibility Criteria

Studies meeting the PICOS inclusion and exclusion criteria presented in Table 1 were included in the review [24].

### 2.3. Information Sources

The Bielefeld Academic Search Engine (BASE), the Cochrane Library (CENTRAL), and PubMed were used to search medical databases. To search sources not indexed in the databases covered by the above-mentioned engines, the Google Scholar engine was used.

### 2.4. Search Strategy

For all engines, the same query “(temporomandibular OR tmj) AND (gas* OR oxygen OR o2 OR ozone OR o3 “air” OR carbon OR co2) AND (“intra-articular” OR intraarticular OR injection* OR arthrography OR arthrocenteses* OR insufflation*)” was used, which was developed based on initial searches using the BASE engine. Reference searches of identified systematic reviews and eligible studies were performed using the backward snowballing method. The final searches were conducted on: (1) 14 October 2025—medical databases; (2) 16 October 2025—Google Scholar; (3) 16 October 2025—references.

### 2.5. Selection Process

Two independent reviewers (M.C. and K.R.) selected titles and abstracts (first stage) and full texts (second stage). In case of discrepancies, decisions were made through discussion, and in case of persistent disagreement, a third reviewer (K.C.) held the final vote. Records were managed using the Rayyan web application [25]. The selection process was visualized using the Shiny web application [26].

### 2.6. Data Collection Process

The following information was extracted from each included article: author, year of publication, number of patients, gas type, administration protocol (volume, frequency, additional procedures), follow-up time, clinical outcomes (pain, mandibular mobility, quality of life over time), and adverse events. Data extraction was performed by two independent reviewers (W.M. and M.C.), and discrepancies were resolved as described above.

### 2.7. Study Risk of Bias Assessment

Methodological quality and risk of bias were assessed by two independent investigators (M.C. and A.H.) using Cochrane RoB-2 (with the same handling of disagreements as above) [27]. To assess the level of inter-reviewer agreement, Cohen’s kappa was calculated using the individual responses to the signaling questions. In accordance with the tool’s recommendations, ‘Yes’ and ‘Probably yes’ were treated as the same response, as were ‘No’ and ‘Probably no’, resulting in four categories: Y/PY (Yes/Probably yes), N/PN (No/Probably no), NA (Not applicable), NI (No information). The results of the risk-of-bias assessment were additionally visualized using the robvis tool (University of Bristol, Bristol, UK; accessed 2 February 2026).

### 2.8. Effect Measures and Synthesis Methods

Only continuous outcomes were analyzed: pain intensity (VAS), jaw mobility (MMO, mm), and quality of life (VAS or %). Quantitative synthesis was limited to the 3-month time point. Effect measures were calculated both within- and between-groups. Specifically: (a) change from baseline was defined as Δ = mean_3 months − mean_baseline; (b) between-group difference (MD) was calculated as the difference in mean final values at month 3.

Pain intensity was assessed using the Visual Analogue Scale (VAS). As the included studies reported VAS results using different formats, all values were standardized to a 0–10 scale to ensure comparability across studies. When necessary, scores originally reported on alternative ranges (e.g., 0–100) were converted proportionally to the 0–10 format.

Quality of life and functional outcomes were reported using different instruments across studies, including VAS-based assessments and the Helkimo Dysfunction Index (DI). Due to the differences in measurement properties between ordinal indices and continuous scales, these outcomes were interpreted descriptively and not directly combined in a pooled statistical analysis.

For between-group comparisons, 95% confidence intervals were calculated based on standard errors calculated from standard deviations and group sizes, assuming independence of observations. Missing means were not imputed; measures of variability were derived exclusively from reported statistics, where possible.

Results were presented as estimates at the individual study level, and plots were used solely to visualize effects without pooling. Intervention was defined as intra-articular ozone-based treatment (O_2_–O_3_ mixture or ozonated solutions), including in combination protocols with arthrocentesis. Multi-arm studies were analyzed as separate comparisons of intervention arms with a control group; changes from baseline were interpreted descriptively.

For transparency, additional assumptions underlying the calculations were specified. Change scores were derived from reported mean values at baseline and at 3 months (Δ = mean_3 months − mean_baseline) without applying paired variance corrections, as individual-level correlation coefficients were not reported. Between-group comparisons were therefore treated as independent observations. In multi-arm trials, each intervention arm was analyzed as a separate comparison with the control group; shared control groups were not statistically adjusted or pooled, and the resulting estimates were interpreted descriptively rather than combined in a meta-analytic model.

### 2.9. Certainty Assessment

The certainty of evidence was assessed using the GRADE approach, which classifies evidence into four levels based on risk of bias, inconsistency, imprecision, and indirectness. Given the heterogeneity of follow-up durations and the limited availability of comparable long-term data, the GRADE assessment was restricted to long-term outcomes (6–12 months), reflecting the durability of treatment effects.

## 3. Results

### 3.1. Study Selection

**Figure 1:**
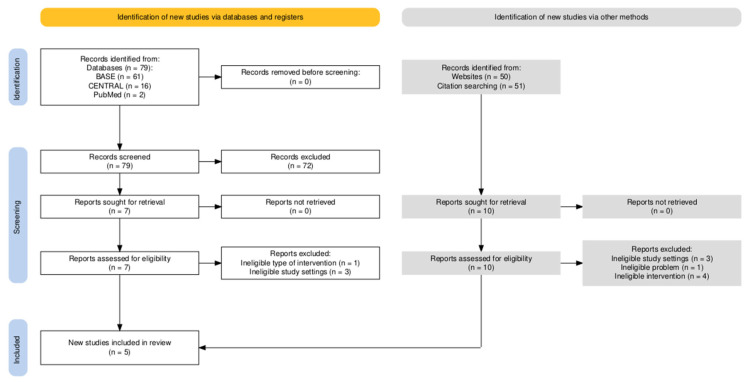
Selection process.

Medical database searches yielded the following number of records depending on the engine: (1) BASE: 61 items; (2) CENTRAL: 16 items; (3) PubMed: 2 items. The screening (with simultaneous deduplication) yielded seven articles that were fully qualified for full-text evaluation (Cohen’s kappa = 0.71). The items from the database selection were supplemented with articles qualified for full-text evaluation based on searches of the first 50 results using the Google Scholar engine. The reference review included primary studies qualified using the above methods and systematic reviews identified using the above methods (Machado et al., Torres-Rosas et al., and de Sire et al.) [9,19,21]. The selection process is illustrated in Figure 1. The results of the full-text evaluation are presented in Table 2.

### 3.2. Included Primary Studies Characteristics

The general characteristics of the included primary studies are presented in Table 3.

The review included five studies from 2012 to 2025, involving 28 to 60 patients, for a total of 230. The ozone–oxygen injections had a volume of 2 mL, with concentrations ranging from 10 to 30 µg/mL. Treatment protocols varied, including single injections and series of up to six treatments, often combining ozone therapy with additional substances such as anesthetics, hyaluronic acid, PRP, and anti-inflammatory medications.

In Bakry’s study, patients were prescribed 600 mg of ibuprofen twice daily for one week, an antibiotic (amoxicillin with clavulanic acid, 1 g twice daily for one week), and a muscle relaxant (multilayer tablets containing orphenadrine citrate, aspirin, and caffeine) twice daily for two weeks as postoperative medication [33].

In the study by Cömert Kiliç (June 2025), patients were prescribed 500 mg of paracetamol three times daily postoperatively [28].

### 3.3. Results of Individual Studies

The change over time in mean outcome values in the domains of joint pain, jaw range of motion, and quality of life in the study and control groups was extracted for Table A1, Table A2 and Table A3.

The findings reported by Bakry (2022) and Khidr (2023) converge with an unusually high degree of numerical similarity [31,33]. Therefore, for the purposes of synthesis, the studies by Bakry (2022) and Khidr (2023) were treated as likely representing the same study, with the two articles considered complementary reports of its outcomes [31,33].

### 3.4. Risk of Bias in Studies

**Figure 2:**
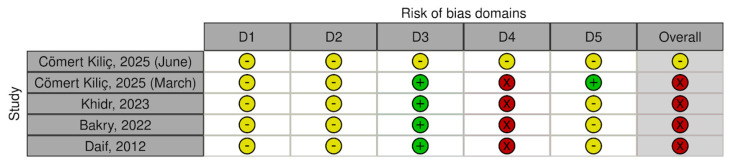
Risk of bias in primary studies [17,18,28,31,33]. Domains: D1—bias arising from the randomization process; D2—bias due to deviations from intended intervention; D3—bias due to missing outcome data; D4—bias in measurement of the outcome; D5—bias in selection of the reported result. Green—low risk; yellow—some concerns; red—high risk. Generated by the authors using the robvis tool.

**Figure 3:**
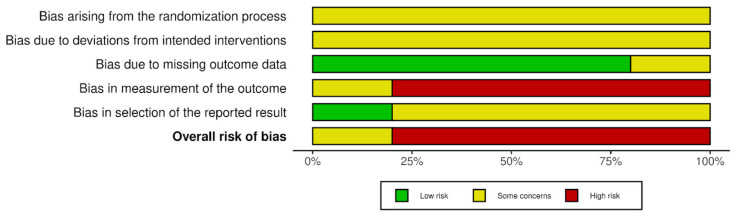
Risk of bias distribution. Generated by the authors using the robvis tool.

In accordance with the RoB2 tool, four studies were judged to be of high risk of bias and one as showing some concerns. The assessment of each study was summarized in Figure 2 and Figure 3, presenting judgements for the individual domains as well as the overall risk of bias judgement. Inter-rater agreement was high, with 86% concordance between ratings and Cohen’s kappa at 0.805, indicating a level of agreement between ranges typically interpreted as substantial agreement (0.61–0.80) and almost perfect agreement (0.81–1.00), with the last one representing the highest possible category of agreement [17,18,28,31,33].

### 3.5. Results of Syntheses

Mean differences from baseline and between groups were calculated and presented (Table 4, Table 5, Table 6 and Table 7).

Analysis of pain intensity at the 3-month follow-up showed improvement compared with baseline in all included patient groups, as confirmed by negative mean differences (MD) for each intervention. The greatest reduction (>7.9) was observed in the Khidr (2023) and Bakry (2022) studies, where ozone therapy (gas or water) was combined with joint irrigation with Ringer’s solution [31,33]. In the Cömert Kılıç (2025) study, different outcomes were reported across the investigated ozone-based intervention arms [18,28].

In each study group, the range of mandibular movement increased over the 3 months, as confirmed by the positive mean difference across all interventions. The greatest improvement, from 17.29 mm to 18.79 mm, was achieved in the Bakry (2022) and Khidr (2023) studies, which combined ozone therapy with Ringer’s solution [31,33]. In the Cömert Kılıç (2025) study, the improvement in movement was weaker, with combined therapy with corticosteroids (CS) proving most effective in this subgroup [18,28]. In neither study did patients achieve the average opening of 40–52 mm.

Comparative analysis of the control groups shows that in all studies, ozone therapy was associated with lower final pain intensity. The study by Cömert Kılıç (2025) reported the largest point differences in favor of the intervention, but the wide confidence intervals obtained suggest a lower significance of the results [18,28]. In contrast, the study by Khidr (2023), despite smaller differences, presents narrow confidence intervals that do not include zero, indicating high precision and significance of the observed therapeutic effect compared to controls [31].

Comparison of the results for mandibular mobility with control groups showed variable findings across studies using different ozone-based protocols, including combinations with arthrocentesis. The highest increase was observed in the study by Khidr (2023) for the group receiving ozone gas with Ringer’s solution [31]. The result for the group receiving ozone water was lower, but the obtained confidence intervals not including zero indicate the high significance of this author’s results. In the study by Cömert Kılıç (2025), the results are characterized by variability [18]. The group treated with a mixture of ozone and anesthetic showed a slight improvement compared to controls, while ozone alone was associated with a worse result than the control group. Furthermore, the obtained results are characterized by wide confidence intervals, suggesting a lack of statistical certainty.

**Figure 4:**
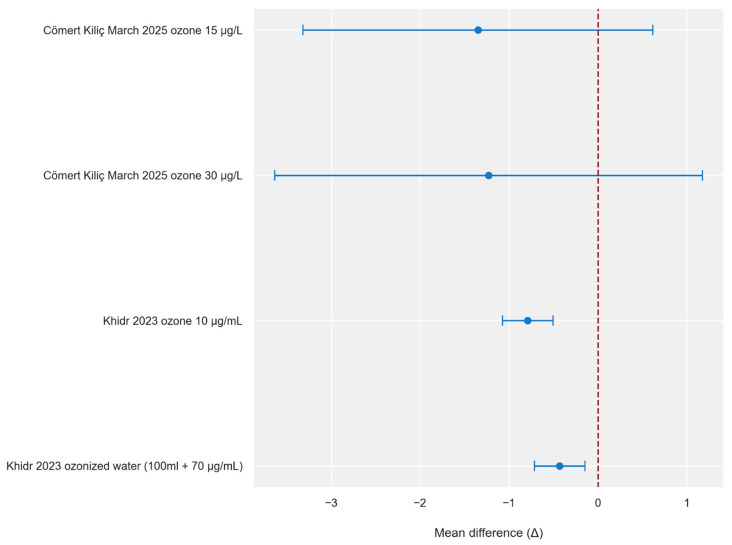
Intergroup difference in TMJ pain intensity (VAS) 3 months following intra-articular ozone treatment (per type of intervention)—visualization of mean differences [18,31]. Negative values (<0) indicate better improvement in the intervention group. The blue circles represent point estimates of mean differences, and the horizontal blue lines represent 95% confidence intervals. The red dashed vertical line indicates no difference between groups (Δ = 0). Δ—change from baseline to 3 months.

**Figure 5:**
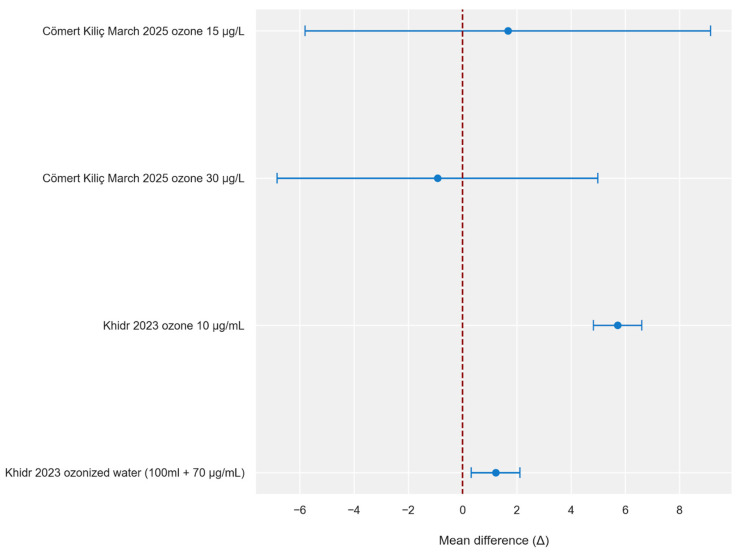
Intergroup difference in mandibular mobility (millimeters) 3 months following intra-articular ozone treatment (per type of intervention)—visualization of mean differences [18,31]. Positive values (>0) indicate greater improvement in the intervention group. The blue circles represent point estimates of mean differences, and the horizontal blue lines represent 95% confidence intervals. The red dashed vertical line indicates no difference between groups (Δ = 0). Δ—change from baseline to 3 months.

Figure 4 and Figure 5 were created based on the obtained results (Table 6 and Table 7). Their graphical analysis clearly highlights the disparity in the precision of the estimates and shows which studies have wide horizontal lines representing confidence intervals that intersect the line of no effect.

### 3.6. Certainty of Evidence

The quality of evidence analysis for long-term follow-up (6–12 months) showed very low certainty for pain intensity, jaw mobility, and quality of life. A detailed summary of the assessment is presented in Table 8.

## 4. Discussion

The included articles describe various therapeutic approaches for temporomandibular joint (TMJ) disorders using medical ozone, applied either as intra-articular injections or in combination with arthrocentesis. Ozone, in concentrations ranging from 10 to 30 μg/mL, was used alone or compared with other substances such as hyaluronic acid, corticosteroids, or ozonated water. Its proposed therapeutic effects are related to anti-inflammatory, analgesic, and regenerative properties that may improve tissue oxygenation and modulate degenerative processes within the joint. All studies aimed to reduce pain, improve mandibular mobility, and restore masticatory function in patients suffering from TMJ osteoarthritis or internal derangements [6,16,18].

All five included studies reported favorable outcomes, although these findings should be interpreted cautiously in view of the methodological limitations of the evidence base. Ozone-based interventions were associated with reductions in pain, increases in maximal mouth opening, and improvement in joint function compared with baseline, with some studies reporting persistence of these effects for up to 1 year. In some individual studies, ozone-based interventions showed more favorable results than pharmacologic treatments (NSAIDs, corticosteroids), hyaluronic acid injections, or simple arthrocentesis; however, these comparisons were study-specific and do not allow firm conclusions regarding superiority or durability of effect. Accordingly, the authors of the included studies described intra-articular ozone application as a potentially useful and minimally invasive approach to TMJ disorders, while also emphasizing the need for further studies and protocol standardization [17,18,28,31,33].

A notable challenge in interpreting the available evidence is the substantial clinical heterogeneity among the included trials. The studies differed in several key aspects, including ozone concentration, number and frequency of injections, and the use of adjunctive procedures such as arthrocentesis or intra-articular administration of other agents (e.g., hyaluronic acid, corticosteroids, or platelet-rich plasma). In addition, variations in concomitant medications and postoperative management further limit direct comparability between studies. This heterogeneity complicates the interpretation of the reported outcomes and makes it difficult to determine the independent contribution of ozone therapy to the observed clinical improvements. Because of the small number of eligible trials and the variability in study designs and protocols, meaningful subgroup or sensitivity analyses (e.g., according to ozone concentration, use of arthrocentesis, or adjunctive therapies) were not feasible. Consequently, the findings of the present review should be interpreted with caution, and future randomized controlled trials should aim to use more standardized treatment protocols and clearer reporting of procedural parameters.

An important aspect in interpreting the available evidence is the distinction between short-term and longer-term outcomes. The most consistent findings were observed at the 3-month follow-up, when patients receiving ozone-based interventions generally showed reduced pain intensity and increased mandibular mobility compared with baseline. In some studies, these short-term outcomes also appeared more favorable than those in the control groups, although the precision of the estimates varied across comparisons and the certainty of evidence remained limited. In contrast, the durability of treatment effects at 6–12 months was uncertain and supported by evidence of very low certainty. While some studies suggested maintenance of improvement, others showed partial loss of the initial benefit over time, particularly with respect to pain intensity. Longer-term patterns for mandibular mobility appeared somewhat more stable, but these findings were also based on limited data and should be interpreted with caution.

The excluded articles broaden the scientific perspective on ozone therapy, demonstrating its applications beyond the temporomandibular joint and across a wide range of musculoskeletal disorders. Seyam et al. (2018) highlighted ozone’s multifaceted mechanisms—promoting tissue regeneration, improving vascularization, and reducing oxidative stress—and presented evidence for its use in osteoarthritis, bursitis, carpal tunnel syndrome, and spinal pain [38]. These findings position ozone as a safe, minimally invasive alternative to pharmacological treatments. It should be noted that most mechanistic insights are derived from experimental and non-TMJ models, and their direct translation to TMJ tissues remains hypothetical.

Other studies explored comparative and broader therapeutic contexts. Raeissadat et al. (2018) compared ozone injections with lidocaine and dry needling for myofascial pain syndrome, reporting that all three methods were beneficial, with ozone and lidocaine slightly outperforming dry needling in short-term outcomes [37].

### 4.1. Clinical Implications

Given the very low certainty of the available evidence and the methodological limitations of the included studies, it is premature to formulate clinical guidelines for the routine use of intra-articular ozone therapy in temporomandibular disorders. At present, this intervention should primarily be considered within controlled clinical settings or research protocols.

From a technical perspective, intra-articular ozone therapy requires dedicated medical ozone generators capable of producing precise and reproducible gas concentrations. Because ozone effects are dose-dependent, strict control of concentration is essential. The procedure also requires sterile intra-articular injection techniques and clinicians trained in accessing the temporomandibular joint. In addition, because ozone is a highly reactive oxidative gas, appropriate safety measures should be ensured during preparation and administration. These include adequate ventilation, controlled gas handling, and equipment designed to minimize ozone leakage and occupational exposure of healthcare personnel.

Practical implementation may be further limited by the lack of standardized treatment protocols. Well-designed randomized controlled trials with clearly defined diagnostic subgroups of TMD and standardized procedural parameters are needed to assess both clinical effectiveness and practical feasibility.

### 4.2. Limitations

This systematic review provides an up-to-date, coherent and valuable analysis of the therapeutic effects of intra-articular ozone injections. It should be emphasized, however, that the number of available studies addressing this topic remains limited, which impacts the strength of conclusions. In addition, the small number of available studies limits the ability to formally assess publication bias, and the possibility that studies with negative or inconclusive results remain unpublished cannot be excluded. Furthermore, five studies assessed using the RoB-2 tool were classified as high risk or raise some concerns about bias, primarily due to ambiguity in the outcome measurement methodology (domain 4). Additional caution should be exercised when interpreting the results because two of the included studies, from different authors, show surprisingly similar outcome values.

A significant source of heterogeneity was also the variety of therapeutic protocols—patients received gaseous ozone at varying concentrations, combined with various substances (including hyaluronic acid, corticosteroids, platelet-rich plasma, Ringer’s solution), or used in the form of ozonated water. It is also worth noting that the literature search was conducted using only English keywords, which creates a risk of omitting studies published in other languages and limits the full representativeness of the collected material.

## 5. Conclusions

This review suggests that intra-articular ozone therapy may be associated with pain reduction and improved jaw mobility in patients with TMJ disorders. However, these findings should be interpreted with caution because of the substantial heterogeneity of the treatment protocols, the risk of bias in the included studies, and the very low certainty of the evidence according to the GRADE assessment. Therefore, the current evidence remains insufficient to support firm conclusions regarding the effectiveness of intra-articular ozone therapy, and further well-designed randomized controlled trials are needed.

## Figures and Tables

**Table 1:** Eligibility criteria.

	Inclusion	Exclusion
Patients	TMD diagnosis	Animal and cadaver studies
Intervention	Injection of gas into the TMJ	Injections without therapeutic intent
Comparison	Intra-TMJ injection of different gas, varying protocols for the same gas, or intra-articular injection of other substance	Not applicable
Outcomes	Pain, mandibular mobility, quality of life	Not applicable
Settings	Randomized clinical trials	Preprints, conference proceedings, book chapters, studies other than randomized clinical trials

**Table 2:** Reports evaluated in full text.

First Author, Publication Year	Identifier	Title	Selection Result
Cömert Kiliç, 2025 (June) [28]	DOI: 10.1016/j.joms.2025.05.009	Is Intra-articular Injection of Hyaluronic Acid, Corticosteroid or Platelet-rich Plasma Following Medical Ozone Superior to Medical Ozone Alone in the Treatment of Temporomandibular Joint Osteoarthritis?	Included
Cömert Kiliç, 2025 (March) [18]	DOI: 10.1016/j.jcms.2025.02.016	Does intra-articular medical ozone therapy applied at different concentrates improve outcomes of temporomandibular joint osteoarthritis? A randomized clinical trial	Included
Machado, 2025 [29]	DOI: 10.1007/s10787-025-01904-w	Protective effects of ozone therapy on cartilage degeneration in temporomandibular joint osteoarthritis	Excluded due to animal model
Vaishnavi, 2025 [30]	DOI: 10.4103/jpbs.jpbs_1832_24	A Prospective Clinical Study Evaluating the Efficacy of Intra-Articular Ozone Therapy for Internal Derangement of the Temporomandibular Joint	Excluded due to no control group
Khidr, 2023 [31]	iscientific.org/wp-content/uploads/2024/02/30-ijcbs-23-24-12-30r.pdf	Tempro Mandibular Joint Internal Derangement: A Comparative Analysis of Ozone Gas and Ozonated Water Following Arthrocentesis	Included
Lopes Sora, 2023 [32]	portaldeperiodicos.unibrasil.com.br/index.php/anaisevinci/article/view/7095	Revisão De Literatura: Ozonioterapia Em Tratamentos Temporomandibulares	Excluded due to being a review article
Bakry, 2022 [33]	DOI: 10.21608/edj.2022.111917.1916	Comparative Evaluation Of Ozone Gas And Ozonated Water After Arthrocentesis In Management Of Tempro Mandibular Joint Internal Derangement: A Clinical Randomized Controlled Study	Included
Haghighat, 2020 [34]	DOI: 10.4103/abr.abr_105_20	Effectiveness of Ozone Injection Therapy in Temporomandibular Disorders	Excluded due to being a review article
Tortelli, 2019 [35]	DOI: 10.1590/1807-2577.10719	Effectiveness of acupuncture, ozonio therapy and low-intensity laser in the treatment of temporomandibular dysfunction of muscle origin: a randomized controlled trial	Excluded due to ineligible intervention
González Rodríguez, 2018 [36]	revactamedicacentro.sld.cu/index.php/amc/article/view/889	Efectividad de la ozonoterapia para disminuir el dolor en los pacientes con trastornos temporomandibulares	Excluded due to non-intra-articular gas administration
Raeissadat, 2018 [37]	DOI: 10.2147/JPR.S164629	Comparison of ozone and lidocaine injection efficacy vs. dry needling in myofascial pain syndrome patients	Excluded due to ineligible intervention
Seyam, 2018 [38]	DOI: 10.4103/2045-9912.241075	Clinical utility of ozone therapy for musculoskeletal disorders	Excluded due to being a review article
Valdes Reyes, 2016 [39]	DOI: 10.4172/2167-0846	Ozone therapy as an alternative treatment to the pain in the temporomandibular disorder	Excluded due to being a conference abstract
Doğan, 2014 [40]	DOI: 10.1159/000365355	Effects of High-Frequency Bio-Oxidative Ozone Therapy in Temporomandibular Disorder-Related Pain	Excluded due to ineligible intervention
Hammuda, 2013 [41]	api.semanticscholar.org/CorpusID:212563898	Use of Ozone in Temporomandibular Joint Arthrocentesis, Clinical Study	Excluded due to ineligible intervention
Daif, 2012 [17]	DOI: 10.1016/j.tripleo.2011.08.006	Role of intra-articular ozone gas injection in the management of internal derangement of the temporomandibular joint	Included
Shallenberger, 2011 [42]	journalofprolotherapy.com/pdfs/issue_10/issue_10_06_prolozone_regenerates_joints.pdf	Prolozone™—Regenerating Joints and Eliminating Pain	Excluded due to ineligible problem

**Table 3:** Study characteristics.

First Author, Publication Year	Number of Patients	Number of Injections	Time Intervals Between Injections	Additives to The Injectable Substance	Follow-Up Time	Adverse Events
Cömert Kiliç, 2025 (June) [28]	52	4	1 week	1 mL intra-articular anesthesia HA, CS, PRP (single session after the initial 4 weeks of injections)	6.09 ± 0.57 months	3 patients dropped out due to lack of checkups
Cömert Kiliç, 2025 (March) [18]	48	4	1 week	1 mL intra-articular anesthesia	13.56 ± 2.08 months	10 patients dropped out due to a lack of checkups
Khidr, 2023 [31]	42	1	-	-	12 months	-
Bakry, 2022 [33]	28	1	-	Ringer’s Solution (100–200 mL)	12 months	-
Daif, 2012 [17]	60	6	3 weeks	nonsteroidal anti-inflammatory drugs and muscles relaxants (only 2. group)	2 weeks	-

**Table 4:** Changes in TMJ pain intensity (VAS) from baseline to 3 months following intra-articular ozone treatment.

First Author, Year	Group (Intervention)	Mean Difference (Δ)	Standard Error (SE)	Lower 95% CI	Upper 95% CI
Cömert Kiliç, 2025 (June) [28]	O_2_–O_3_ 15 µg/L, 2 mL	−3.520	1.304	−6.2116	−0.8284
Cömert Kiliç, 2025 (June) [28]	O_2_–O_3_ 15 µg/L, 2 mL 1 mL HA	−5.930	1.060	−8.1282	−3.7318
Cömert Kiliç, 2025 (June) [28]	O_2_–O_3_ 15 µg/L, 2 mL 1 mL CS	−4.920	1.064	−7.1258	−2.7142
Cömert Kiliç, 2025 (June) [28]	O_2_–O_3_ 15 µg/L, 2 mL 1 mL PRP	−4.410	1.076	−6.6407	−2.1793
Cömert Kiliç, 2025 (March) [18]	O_2_–O_3_ 15 µg/L, 2 mL + 4% articaine, adrenaline 1:100 000, 1 mL	−4.750	0.870	−6.5277	−2.9723
Cömert Kiliç, 2025 (March) [18]	O_2_–O_3_ 30 µg/L, 2 mL	−3.320	1.288	−6.0062	−0.6338
Bakry, 2022 [33] and Khidr, 2023 [31]	Ringer’s Solution (100–200 mL) + ozone (10 µg/mL, 2 mL)	−8.430	0.091	−8.6179	−8.2421
Bakry, 2022 [33] and Khidr, 2023 [31]	Ringer’s solution (100–200 mL) + ozonized water (100 mL + 70 µg/mL)	−7.930	0.091	−8.1179	−7.7421

VAS—visual analogue scale; Δ—change from baseline to 3 months; SE—standard error; CI—confidence interval; TMJ—temporomandibular joint; 3-month—assessment performed approximately 12 weeks after baseline measurement.

**Table 5:** Changes in mandibular mobility (millimeters) from baseline to 3 months following intra-articular ozone treatment.

First Author, Year	Group (Intervention)	Mean Difference (Δ)	Standard Error (SE)	Lower 95% CI	Upper 95% CI
Cömert Kiliç, 2025 (June) [28]	O_2_–O_3_ 15 µg/L, 2 mL	7.700	2.646	2.2393	13.1607
Cömert Kiliç, 2025 (June) [28]	O_2_–O_3_ 15 µg/L, 2 mL 1 mL HA	9.420	2.513	4.2081	14.6319
Cömert Kiliç, 2025 (June) [28]	O_2_–O_3_ 15 µg/L, 2 mL 1 mL CS	11.500	2.095	7.1561	15.8439
Cömert Kiliç, 2025 (June) [28]	O_2_–O_3_ 15 µg/L, 2 mL 1 mL PRP	8.500	3.029	2.2183	14.7817
Cömert Kiliç, 2025 (March) [18]	O_2_–O_3_ 15 µg/L, 2 mL + 4% articaine, adrenaline 1:100 000, 1 mL	5.180	3.301	−1.5616	11.9216
Cömert Kiliç, 2025 (March) [18]	O_2_–O_3_ 30 µg/L, 2 mL	5.790	3.414	−1.3318	12.9118
Bakry, 2022 [33] and Khidr, 2023 [31]	Ringer’s Solution (100–200 mL) + ozone (10 µg/mL, 2 mL)	18.290	0.563	17.1320	19.4480
Bakry, 2022 [33] and Khidr, 2023 [31]	Ringer’s solution (100–200 mL) + ozonized water (100 mL + 70 µg/mL)	17.290	0.463	16.3373	18.2427

Δ—change from baseline to 3 months; SE—standard error; CI—confidence interval; 3-month—assessment performed approximately 12 weeks after baseline measurement.

**Table 6:** Intergroup difference in TMJ pain intensity (VAS) 3 months following intra-articular ozone treatment.

First Author, Year	Mean Difference (Δ)	Standard Error (SE)	Lower 95% CI	Upper 95% CI
Cömert Kiliç, 2025 (March), ozone 15 µg/L [18]	−1.350	0.956	−3.3198	0.6198
Cömert Kiliç, 2025 (March), ozone 30 µg/L [18]	−1.230	1.153	−3.6355	1.1755
Khidr, 2023, ozone 10 µg/mL [31]	−0.790	0.138	−1.0736	−0.5064
Khidr, 2023, ozonized water (100 mL + 70 µg/mL) [31]	−0.430	0.138	−0.7136	−0.1464

VAS—visual analogue scale; Δ—change from baseline to 3 months; SE—standard error; CI—confidence interval; TMJ—temporomandibular joint; 3-month—assessment performed approximately 12 weeks after baseline measurement.

**Table 7:** Intergroup difference in mandibular mobility (millimeters) 3 months following intra-articular ozone treatment.

First Author, Year	Mean Difference (Δ)	Standard Error (SE)	Lower 95% CI	Upper 95% CI
Cömert Kiliç, 2025 (March), ozone 15 µg/L [18]	1.670	3.628	−5.8030	9.1430
Cömert Kiliç, 2025 (March), ozone 30 µg/L [18]	−0.920	2.835	−6.8327	4.9927
Khidr, 2023, ozone 10 µg/mL [31]	5.720	0.435	4.8265	6.6135
Khidr, 2023, ozonized water (100 mL + 70 µg/mL) [31]	1.220	0.437	0.3226	2.1174

Δ—change from baseline to 3 months; SE—standard error; CI—confidence interval; 3-month—assessment performed approximately 12 weeks after baseline measurement.

**Table 8:** GRADE quality of evidence assessment (long-term outcomes).

Outcome	No. of Participants (Studies)	Certainty of Evidence (GRADE)	Comments
Pain Intensity	90-142 * (3 RCTs)	Very low	Risk of Bias: Downgraded by 2 levels due to high risk of bias in most included studies. Inconsistency: Downgraded by 1 level due to differences in the durability of the effect. The Khidr study reported recurrence of pain (increase from 1.57 to 3.79 at 12 months), whereas the Cömert Kiliç study showed maintenance of improvement [18,31]. Imprecision: Downgraded by 1 level due to the small sample size (*n* < 400).
MMO	90-142 * (3 RCTs)	Very low	Risk of Bias: Downgraded by 2 levels due to high risk of bias in most included studies. Inconsistency: Downgraded by 1 level due to mixed results and wide confidence intervals. Imprecision: Downgraded by 1 level due to the small sample size.
Quality of Life	48 1 RCT	Very low	Risk of Bias: Downgraded by 1 level due to high risk of bias in the included study. Imprecision: Downgraded by 2 levels due to the critically small sample size and the fact that only one study (Cömert Kiliç March) reported 12-month outcomes. The lack of data from other studies prevents a reliable assessment [18].

*—The number of participants is approximately 170 at month 6 (4 studies), but decreases to 118 at month 12 because the Cömert Kiliç study was ending at the 6-month follow-up.

## Data Availability

No new data were created or analyzed in this study. Data sharing does not apply to this article.

## Abbreviations

The following abbreviations are used in this manuscript:

ARE	Antioxidant Response Element
BASE	Bielefeld Academic Search Engine
CENTRAL	Cochrane Central Register of Controlled Trials
CI	Confidence Interval
CS	Corticosteroids
DI	Dysfunction Index
GRADE	Grading of Recommendations Assessment, Development and Evaluation
HA	Hyaluronic Acid
HO-1	Heme Oxygenase-1
IL-1β	Interleukin-1 beta
IL-6	Interleukin-6
Keap1	Kelch-like ECH-associated protein 1
LOPs	Lipid Oxidation Products
MD	Mean Difference
MIO	Maximal Interincisal Opening
MMO	Maximal Mouth Opening
mm	millimeters
MMPs	Matrix Metalloproteinases
MMP-3	Matrix Metalloproteinase-3
MMP-13	Matrix Metalloproteinase-13
mTOR	mechanistic Target Of Rapamycin
NF-κB	Nuclear Factor kappa-light-chain-enhancer of activated B cells
NSAIDs	Nonsteroidal Anti-Inflammatory Drugs
Nrf2	Nuclear factor erythroid 2–related factor 2
O_2_–O_3_	Oxygen–Ozone mixture
PICOS	Population, Intervention, Comparison, Outcomes, Study design
PPARγ	Peroxisome Proliferator-Activated Receptor gamma
PRISMA	Preferred Reporting Items for Systematic Reviews and Meta-Analyses
PROSPERO	International Prospective Register of Systematic Reviews
PRP	Platelet-Rich Plasma
RCT	Randomized Controlled Trial
RDC/TMD	Research Diagnostic Criteria for Temporomandibular Disorders
RoB-2	Cochrane Risk of Bias Tool, version 2
RONS	Reactive Oxygen and Nitrogen Species
SD	Standard Deviation
SE	Standard Error
SOD	Superoxide Dismutase
TMDs	Temporomandibular Disorders
TMJ	Temporomandibular Joint
TNF-α	Tumor Necrosis Factor alpha
VAS	Visual Analog Scale

## Appendix A

**Table A1:** Pain measures reported in the included studies.

First Author, Publication Year	Patient Group	Baseline Value	1 Month	3 Months	6 Months	9 Months	12 Months
Cömert Kiliç, 2025 (June) [28]	O_2_–O_3_ 15 µg/L, 2 mL	5.75 ± 3.50	-	2.23 ± 3.14	1.57 ± 2.49	-	-
Cömert Kiliç, 2025 (June) [28]	O_2_–O_3_ 15 µg/L, 2 mL 1 mL HA	7.05 ± 3.16	-	1.12 ± 1.87	2.74 ± 4.22	-	-
Cömert Kiliç, 2025 (June) [28]	O_2_–O_3_ 15 µg/L, 2 mL 1 mL CS	7.61 ± 2.26	-	2.69 ± 2.91	2.47 ± 2.28	-	-
Cömert Kiliç, 2025 (June) [28]	O_2_–O_3_ 15 µg/L, 2 mL 1 mL PRP	6.98 ± 2.21	-	2.57 ± 3.00	1.16 ± 2.48	-	-
Cömert Kiliç, 2025 (March) [18]	4% articaine, adrenaline 1:100 000, 1 mL	4.72 ± 2.09	-	3.51 ± 2.11	-	-	1.91 ± 1.54
Cömert Kiliç, 2025 (March) [18]	O_2_–O_3_ 15 µg/L, 2 mL + 4% articaine, adrenaline 1:100 000, 1 mL	6.91 ± 2.27	-	2.16 ± 2.64	-	-	1.47 ± 2.47
Cömert Kiliç, 2025 (March) [18]	O_2_–O_3_ 30 µg/L, 2 mL + 4% articaine, adrenaline 1:100 000, 1 mL	5.60 ± 2.84	-	2.28 ± 3.19	-	-	0.80 ± 1.62
Khidr, 2023 [31]	Ringer’s solution	9.35 ± 0.20	1.00 ± 0.36	1.50 ± 0.44	1.96 ± 0.49	2.64 ± 0.39	3.64 ± 0.50
Khidr, 2023 [31]	Ringer’s Solution + ozone 10 µg/mL, 2 mL	9.14 ± 0.21	1.86 ± 0.70	0.71 ± 0.27	0.43 ± 0.14	0.71 ± 0.27	1.21 ± 0.58
Khidr, 2023 [31]	Ringer’s solution + ozonized water (100 mL + 70 µg/mL)	9.00 ± 0.21	0.57 ± 0.20	1.07 ± 0.27	1.57 ± 0.36	2.64 ± 0.37	3.79 ± 0.47
Bakry, 2022 [33]	Ringer’s Solution (100–200 mL) + ozone (10 µg/mL, 2 mL)	9.14 ± 0.21	1.86 ± 0.70	0.71 ± 0.27	0.43 ± 0.14	0.71 ± 0.34	1.21 ± 0.58
Bakry, 2022 [33]	Ringer’s solution (100–200 mL) + ozonized water (100 mL + 70 µg/mL)	9.00 ± 0.21	0.57 ± 0.20	1.07 ± 0.27	1.57 ± 0.36	2.64 ± 0.37	3.79 ± 0.47

Data are presented as mean ± standard deviation for each group at the respective time point. Values are given in centimeters of 10 cm visual analog scale.

**Table A2:** Mandibular mobility measures reported in the included studies.

First Author, Publication Year	Patient Group	Baseline Value	1 Month	3 Months	6 Months	9 Months	12 Months
Cömert Kiliç, 2025 (June) [28]	O_2_–O_3_ 15 µg/L, 2 mL	20.92 ± 6.66	-	28.62 ± 6.83	32.85 ± 5.70	-	-
Cömert Kiliç, 2025 (June) [28]	O_2_–O_3_ 15 µg/L, 2 mL 1 mL HA	24.50 ± 6.42	-	33.92 ± 5.88	31.25 ± 7.16	-	-
Cömert Kiliç, 2025 (June) [28]	O_2_–O_3_ 15 µg/L, 2 mL 1 mL CS	24.00 ± 4.79	-	35.50 ± 5.45	32.92 ± 4.62	-	-
Cömert Kiliç, 2025 (June) [28]	O_2_–O_3_ 15 µg/L, 2 mL 1 mL PRP	28.17 ± 8.79	-	36.67 ± 5.73	37.25 ± 4.33	-	-
Cömert Kiliç, 2025 (March) [18]	4% articaine, adrenaline 1:100 000, 1 mL	32.47 ± 4.81	-	31.33 ± 6.83	-	-	34.20 ± 5.17
Cömert Kiliç, 2025 (March) [18]	O_2_–O_3_ 15 µg/L, 2 mL	27.82 ± 7.90	-	33.00 ± 10.58	-	-	33.50 ± 8.56
Cömert Kiliç, 2025 (March) [18]	O_2_–O_3_ 30 µg/L, 2 mL	24.62 ± 9.30	-	30.41 ± 6.46	-	-	34.17 ± 5.25
Khidr, 2023 [31]	Ringer’s solution	17,86 ± 1.09	33.50± 1.25	34.07± 1.14	33.21± 1.21	30.86 ± 1.09	28.93± 1.24
Khidr, 2023 [31]	Ringer’s Solution + ozone 10 µg/mL, 2 mL	21.0± 1.76	38.43± 1.12	39.79± 1.16	39.36± 1.32	37.64 ± 1.70	36.36± 2.13
Khidr, 2023 [31]	Ringer’s solution + ozonized water (100 mL + 70 µg/mL)	18.00± 1.28	35.36± 1.16	35.29± 1.17	33.79± 1.12	31.36 ± 1.09	29.14± 1.22
Bakry, 2022 [33]	Ringer’s Solution (100–200 mL) + ozone (10 µg/mL, 2 mL)	21.50 ± 1.76	38.43 ± 1.12	39.79 ± 1.16	39.36 ± 1.32	37.64 ± 1.70	36.36 ± 2.13
Bakry, 2022 [33]	Ringer’s solution (100–200 mL) + ozonized water (100 mL + 70 µg/mL)	18.00 ± 1.28	35.36 ± 1.16	35.29 ± 1.17	33.79 ± 1.12	31.36 ± 1.09	29.14 ± 1.22

Data are presented as mean ± standard deviation for each group at the respective time point. Values are given in millimeters of maximal interincisal mouth opening.

**Table A3:** Quality-of-life measures reported in the included studies.

First Author, Publication Year	Patient Group	Scale Used	Baseline Value	14 Days	3 Months	6 Months	12 Months
Cömert Kiliç, 2025 (March) [18]	4% articaine, adrenaline 1:100 000, 1 mL	VAS	7.69 ± 2.31	-	7.83 ± 2.27	-	8.39 ± 1.80
Cömert Kiliç, 2025 (March) [18]	O_2_–O_3_ 15 µg/L, 2 mL	VAS	6.11 ± 2.08	-	6.69 ± 1.97	-	8.56 ± 2.05
Cömert Kiliç, 2025 (March) [18]	O_2_–O_3_ 30 µg/L, 2 mL	VAS	5.57 ± 1.18	-	7.82 ± 1.89	-	9.17 ± 0.65
Daif, 2012 [17]	O_2_–O_3_ 10 µg/L, 2 mL	DI 0	0%	18.3%	-	-	-
Daif, 2012 [17]	O_2_–O_3_ 10 µg/L, 2 mL	DI I	3.3%	8.3%	-	-	-
Daif, 2012 [17]	O_2_–O_3_ 10 µg/L, 2 mL	DI II	16.67%	20%	-	-	-
Daif, 2012 [17]	O_2_–O_3_ 10 µg/L, 2 mL	DI III	30%	3.3%	-	-	-
Daif, 2012 [17]	400 mg ibuprofen + 250 mg chlorzoxazone	DI 0	0%	0%	-	-	-
Daif, 2012 [17]	400 mg ibuprofen + 250 mg chlorzoxazone	DI I	1.67%	11.67%	-	-	-
Daif, 2012 [17]	400 mg ibuprofen + 250 mg chlorzoxazone	DI II	23.3%	21.67%	-	-	-
Daif, 2012 [17]	400 mg ibuprofen + 250 mg chlorzoxazone	DI III	25%	16.67%	-	-	-

DI—Dysfunction index. In Cömert Kiliç, 2025 (March) VAS was used to assess masticatory efficiency [18]. In Daif, 2012 clinical manifestations were recorded according to the dysfunction index of Helkimo [17]. The patients assigned a score of 0–5 in each item and then the dysfunction index was determined by summation of all points of the Items: DI 0 (0 points) means free of dysfunction symptoms, DI I (1–4 points) means mild dysfunction, DI II (5–9 points) means moderate dysfunction and DI III (10–25 points) means severe dysfunction. Values given in the study were converted according to number of patients included, 100% meaning 60 patients.
